# HBsAg seroprevalence among Senegalese militaries

**DOI:** 10.1186/s40779-015-0032-7

**Published:** 2015-02-24

**Authors:** Abdoul A Ndiaye, Ibrahima Socé Fall, Gora Lo, Sidy Mouhamed Seck, Alioune Badara Tall, Boubacar Gueye, Amady Barro Mbodj, Anta Tal-Dia

**Affiliations:** Community Health Department, University Alioune Diop of Bambey, Bambey City, Senegal; Army Health Service, Camp Dial Diop Dakar, Dakar, Senegal; Faculty of Health Sciences, Gaston Berger University, Saint-Louis, Senegal; Public Health Department, Cheikh Anta Diop University, Dakar, Senegal; World Health Organization, Bamako, Mali

**Keywords:** Seroprevalence, HBsAg, Military, Chronic diseases

## Abstract

**Background:**

Chronic hepatitis is a major public health problem. Hepatitis B virus is the primary cause, and Hepatitis B and C together are responsible for 60% of cirrhosis and 80% of hepatocellular carcinomas. This study measured the prevalence of HBsAg among Senegalese military to develop an appropriate strategy to prevent cirrhosis and hepatocellular carcinoma.

**Methods:**

We conducted a descriptive cross-sectional study among Senegalese military aged 25 to 60 years. A sample of 1224 participants was selected following a two-level-stratification. The mark of surface HBs antigen using chemiluminescence concerned 1195 participants. The presence of HBsAg was analyzed according to age, marital status, alcohol consumption and glomerular filtration rate. Epi-info6fr and R software were used, respectively, for data capture and analyses. A Chi-square test was performed to compare proportions considering a significance level of 5% and a confidence interval of 95%.

**Results:**

The average age was 39.8 ± 9.2 years. Participants in the age groups of 25–34 years, 45–60 years and 35–44 years were respectively 30.7%, 34.4% and 34.9% of the sample. Married persons represented 82.6% of participants and 17.08% were single. Most participants were educated (99%), and 56% had reached at least secondary school level. Alcohol consumption was at 11.5%. The HBsAg prevalence rate was 10.8% [9.1% to 12.7%] with a significant difference between age groups (P < 0.001), which ranged from 5.6% for 45–60 years, 9.62% for 25–34 years to 16.9% for 35–44 years. Marital status and alcohol consumption did not affect the carriage of HBsAg. HBsAg prevalence was more common among participants who had a glomerular filtration rate greater than 90 ml/min. Transaminases rate exceeded the normal threshold for 43 participants (3.6%); the increase was 6.6% [2.7% to 11.8%] for HBsAg carriers and 3.2% [1.2% to 6.7%] for alcohol users.

**Conclusions:**

The high prevalence of HBsAg in the military requires the implementation of an effective prevention and care program to reduce the risk of cirrhosis and hepatocellular carcinoma and contribute to reducing the burden of communicable diseases, such as hepatitis and HIV/AIDS, and non-communicable diseases.

## Background

Chronic viral hepatitis is a worldwide major public health problem, particularly in developing countries that are facing a range of barriers to contain the epidemic [1-5]. Approximately 9% of the world population is suffering from chronic hepatitis. Lemoine et al. [1] reported that 350–400 million of the 550 million people who were infected were infected by the hepatitis B virus (HBV), 170–180 million by the hepatitis C virus (HCV) and 15 million were due to the hepatitis D virus (HDV) [1,6-8]. The poorest regions are the most affected [1,9]. HBV and HCV are responsible for 60% of cirrhosis and 80% of hepatocellular carcinomas, with more than one million deaths annually, mainly in disadvantaged areas [1,6,8,10]. These figures illustrate the role of hepatitis B, which are also confirmed by other authors. The WHO estimates that 2 billion people are infected with HBV worldwide and approximately 380 million are chronic carriers (6% of the world population). Approximately 4.5 million new infections occur annually, and 620,000 deaths due to HBV are reported worldwide [1,11]. The prevalence of HBV is estimated between 8%-15% in endemic areas, such as Africa [1,12,13]. The prevalence in the general population in Senegal was estimated as 11% in 2012 according to the Ministry of Health. One study of pregnant women at the Military Hospital of Ouakam, between 2006 and 2009, showed a prevalence of HBV of 11.6% [14], which indicates the importance of the presence of the virus in the Senegalese general population.

This high prevalence justifies the implementation of a national program against hepatitis and the integration of the vaccine in the Expanded Immunization Program.

The fight against viral hepatitis B is not well structured in the army. The current situation of infection prevalence is unknown. Therefore, one of the objectives of this study was to determine the prevalence of HBsAg in the military to develop strategies for cirrhosis and hepatocellular carcinoma prevention.

## Methods

We performed a descriptive and analytical cross-sectional study in the Senegalese military in the period from May 2013 to February 2014.

Military aged men 25 to 60 years who were present in the country during the survey were included in the study.

Soldiers who had less than 2 years of duty in the military or were unable to answer the questions were excluded.

The enrolled population was divided into three (3) age groups: 25–34, 35–44 and 45–60 years. The survey was conducted in all Senegalese regions.

The sample size was calculated in the framework of STEPS investigation in the military, which provided an estimate of 1,296 men who were divided into three strata. Each member was consistently linked to only one training unit. Therefore, we proceeded with a first selection to determine the number of respondents in each training unit using the human resources central database. A second simple random sampling was performed from the local unit file to identify participants for each stratum.

Information regarding the socio-demographic (age, gender, ethnicity, marital status, educational level, ranks…) and behavioral characteristics (alcohol) of the subjects were collected using a revised STEPS questionnaire [15].

For each participant, 5 ml of venous blood taken from the elbow crease were collected in a dry test tube and centrifuged. Sera were decanted and maintained at 4°C if the analyses were not performed within 4 hours.

HBsAg was initially detected using the DETERMINE HBs Agkit ® (Alere) and ARCHITECT HBsAg QUANTITATIVE II (Abbott Diagnostics) ®. In a second step, measurement of transaminases (AST and ALT) was performed using the controller from Biochemistry Cobas400 ® of Roche Diagnostic laboratories and the Spectrophotometer CYPRESS diagnostic ®.

Chronic renal disease was defined by a glomerular filtration rate expressed in ml per min. The assessment of the glomerular filtration rate (GFR) was performed using the Modification of Diet in Renal Disease (MDRD) formula [16]. Two stages were identified: Stage 1 represented a GFR value greater than 90 ml/min and Stage 2 a GFR between 60 and 90 ml / min [17,18].

Biochemical results were reported on the questionnaire.

Data were entered in Epi-info6 software and analyzed using R software. The Chi-square test was performed to compare proportions with 5% for the significance level and 95% for the confidence interval (CI).

Participation was voluntary. Each participant signed a consent letter at the beginning of the investigation and had the opportunity to stop participating at any stage of the process. All results were returned to the subjects, and participants who were detected as HBs antigen carriers were referred to specialists for treatments.

## Results

Instead of the 1,296 participants who were planned to be involved in the survey, 1,224 soldiers were enrolled, and 1,195 were tested for HBsAg and transaminase levels. The lack of HBsAg research for 29 participants was related to the mobility of soldiers and stock out of test, while not their status.

The mean age was 39.8 years, and it was equal to the median (normal distribution). The sample consisted mostly of men with a sex ratio of 31.2, which followed the personnel structure in the armed forces. Soldiers were more represented (48.0%), followed by non-commissioned officers (45.6%) and officers (6.4%). Most participants were married (82.6%), singles represented 17.08% and other 0.32% of the sample. Serere (25.0%) and Ouolof (24.3%) ethnic groups were more frequent, followed by Diola (18.3%) and Pulaar (16.3%). Almost all respondents were literate (99.1%), and 56.0% had at least achieved secondary level at school (See Table 1).

**Table 1:** **Demographic variables of respondents**

**Item**	**Number**	**Percentage**	**CI**_**95%**_
**Age group**			
25-34years	376	30.71%	[28.1%-33.4%]
35-44years	421	34.40%	[31.7%-37.1%]
45-60years	427	34.89%	[32.2%-37.6%]
**Sex**			
Male	1186	96.90%	[95.7%-97.8%]
Female	38	3.10%	[2.2%-4.3%]
**Professional category**			
Soldiers	588	48.04%	[45.2%-50.8%]
Non-commissioned officers	558	45.59%	[42.8%-48.5%]
Officers	78	6.37%	[5.1%-7.9%]
**Marital status**			
Married	1011	82.60%	[80.4%-84.7%]
Single	209	17.08%	[15.0%-19,3%]
Other	4	0.32%	
**Ethnic group**			
Serere	306	25.0%	[22.6%-27.5%]
Ouolof	297	24.3%	[21.9%-26.8%]
Diola	224	18.3%	[16.2%-20.6%]
Pulaar	200	16.3%	[14.3%-18.5%]
Mandingue	62	5.1%	[3.9%-6.4%]
Other	135	11.0%	[9.3%-12.9%]
**Level of education**			
No education	12	0.9%	[0.5%-1.7%]
Elementary Level	194	15.8%	[13.8%-18.0%]
Average	313	25.6%	[23.1%-28.1%]
High school	565	46.2%	[43.3%-49.0%]
University	140	11.4%	[9.7%-13.4%]

The GFR identified two stages of chronic kidney disease (CKD) among participants: it was higher than 90 ml per min for 68% of participants (CKD stage 1) and between 60 and 90 ml per min for 32% (CKD stage 2).

**Figure 1:**
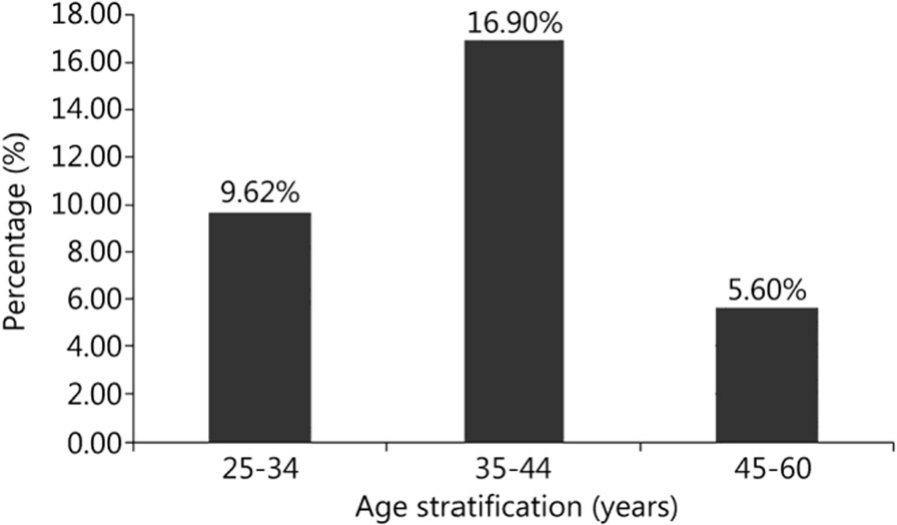
**Prevalence rate of HBsAg carriers according to age group among tested Senegalese soldiers.**

The prevalence of HBsAg was estimated at 10.8% [9.1% to 12.7%], and it varied significantly between the different age groups (P < 0.001). It was 9.62% [6.8% to 13.1%], 16.90% [13.4% to 20.8%] and 5.60% [3.6% to 8.3%] for the age groups 25–34, 35–44 and 45–60 years, respectively (see Figure 1).

Table 2 shows that the carriage of HBsAg was not influenced by ethnic group, marital status and alcohol consumption (P > 0.05). However, it was significantly higher among participants who were classified as GFR stage 1 (12.3%) compared to GFR stage 2 (7.7%) with a p-value equal to 0.023. Similarly, the frequency was different according to rank and was greater for the soldiers (13.6%) than non-commissioned officers (7.9%).

**Table 2:** **HBsAgseroprevalence according to certain socio-behavioral characteristics and chronic kidney disease in the military**

**Item**	HBsAg positive*			
	**Case**	**Percentage (%)**	**CI**_**95%**_	***P*** **value**
**Ethnic group**				
Serere	38	12.6%	[9.1%-16.9%]	0.68
Pulaar	22	11.3%	[7.2%-16.7%]	
Diola	23	10.5%	[6.7%-15.3%]	
Ouolof	24	8.4%	[5.4%-12.2%]	
Mandingue	6	9.8%	[3.7%-20.2%]	
Other	16	12.03%	[7.0%-18.8%]	
**Professional category**				
Soldiers	78	13.6%	[10.9%-16.7%]	0.0087
Non-commissioned officers	43	7.9%	[5.8%-10.5%]	
Officers	8	10.8%	[4.8%-20.2%]	
**Marital status**				
Single	22	11.1%	[7.1%-16.3%]	0.99
Married & other	107	10.7%	[8.9%-12.8%]	
**Alcohol consumption**				
Yes	22	11.8%	[7.6%-17.4%]	0.71
No	107	10.6%	[8.8%-12.7%]	
**Chronic kidney disease: GFR**				
60-90 ml/min	30	7.7%	[5.3%-10.8%]	0.023
>90 ml/min	99	12.3%	[10.1%-14.8%]	

*29 participants absent of HBsAg research.

Transaminase rates were higher than the normal threshold in 43 participants (3.6%); it was 6.6% [2.7% to 11.8%] among carriers of HBsAg and 3.2% [1.2% to 6.7%] among drinkers. However, an increase two times greater than normal was exceptional.

## Discussion

HBsAg prevalence is a public health problem, especially in sub-Saharan Africa [5,19]. An area is classified as a highly endemic area of hepatitis B [20,21] when HBsAg seroprevalence is higher than 8% for adults. Kiire et al. estimated the prevalence of hepatitis B in sub-Saharan Africa between 9% and 12% [19], and the results of this study confirmed that estimate. Indeed, the prevalence of HBsAg was 10.8%, which shows the high endemicity of hepatitis B among Senegalese troops. This result correlated with the observed prevalence in the general population (11%) in 2012 according to the Ministry of Health [22,23], but it is lower than the prevalence among men (21%) stated by Lo et al. in 2014 [23]. The difference in the figures may be due to different target groups these studies on HBsAg. Our study targeted subjects who were apparently in good health, but the study conducted by Gora was based on sick patients who were admitted to the laboratory for further investigation. The current practice in Senegal is the performance of exploratory tests to confirm a clinical diagnosis. Therefore, there may be an overestimation of the prevalence compared to the general population. In fact, the observed prevalence of HBsAg in pregnant women (11.57%), who are representative of the general population, corroborates this hypothesis [14].

A study in Nigeria estimated an HBV seroprevalence 14.6% with a higher frequency in the 20-30-year-old age group [20]. Our study showed a slightly lower prevalence with a higher frequency among 35–44 year-olds. Other authors had already reported a prevalence of HB antigens that was higher among young adults aged 20 to 30 years [14,20]. The hypothesis stated for this finding was that there were more frequent sexual activities among young people. In our study, participants aged 35–44 years were more affected. The decreased prevalence rate in the elders might be related to the impact of condom use campaigns and the extensive ongoing National Hepatitis Program.

The military is considered a gateway group for sexually transmitted diseases (STDs) and HIV/AIDS. The observed prevalence of HBsAg in this study confirms the results of the national survey of combined HIV surveillance in the military, which had shown prevalence figures identical to the general population [24].

HBsAg seroprevalence was 12.3% and 7.7% in participants with CKD stage 1 and stage 2, respectively. Diouf et al. [25] reported an HBsAg seroprevalence (6.7%) among patients receiving dialysis at Aristide Le Dantec Hospital in Dakar (Senegal) that was comparable to our patients with CKD stage 2.

Some authors attempted to link the carrying of HBsAg and CKD [26-30]. In our study, 23.3% of patients with HBsAg were CKD stage 2 versus 33.6% in patients who were seronegative. These results need further investigation in a cohort or case–control study to elucidate the causality.

### Limitations

This study was conducted among soldiers who were selected through incorporation screening based on comprehensive clinical data that satisfied medical fitness criteria to serve in the armed forces. However, this medical screening did not include liver function tests. Accordingly, symptomatic carriers should have been eliminated. In addition, the sample was essentially composed of men. The cross-sectional nature of the study did not allow a demonstration of a causal relationship

## Conclusion

The highly endemic HBsAg prevalence mentioned in this study is similar to the prevalence in the general population. This finding makes sense because no specific measures were taken to expect a different situation until now. The detection of HBsAg is not yet introduced into the army incorporation screening.

The interest of this work was to promote the introduction of Hepatitis B prevention in the overall fight against non-communicable diseases in the army program and communicable diseases, such as HIV/AIDS and hepatitis. Conducting awareness campaigns among the military, periodic screening, improving access to treatment and the training of health personnel will be the major components of this program, which seeks to reduce cirrhosis and hepatocellular carcinoma morbidity and mortality and the burden of non-communicable diseases.

## Abbreviations

CI: Confidence interval
CKD: Chronic kidney disease
GFR: Glomerular filtration rate
HBV: Hepatitis B virus
HCV: Hepatitis C virus
HDV: Hepatitis D virus
MDRD: Modification of Diet in Renal Disease
STDs: Sexually transmitted diseases
